# 942 Partnering Child Life Specialists with Burn Nurses to Improve the Patient Experience of Pediatric Procedures

**DOI:** 10.1093/jbcr/iraf019.473

**Published:** 2025-04-01

**Authors:** Emily Roach, Lindsey Harris, Jasmin Mercedes, Carey Lamphier, Yuk Ming Liu, Laura Johnson, Lauren Nosanov

**Affiliations:** Walter L. Ingram Burn Center at Grady Memorial Hospital; Walter L. Ingram Burn Center at Grady Memorial Hospital; Walter L. Ingram Burn Center at Grady Memorial Hospital; Walter L. Ingram Burn Center at Grady Memorial Hospital; Emory University - Walter L. Ingram Burn Center; Walter L. Ingram Burn Center at Grady Memorial Hospital; Emory University School of Medicine

## Abstract

**Introduction:**

Nurses specializing in burn care face unique challenges when working with pediatric patients. Our metropolitan burn center sees over 600 patients per year, 22-25% of which are under age 18, yet many nurses report a lack of confidence and knowledge of how to approach bedside pediatric procedures. A multidisciplinary initiative was created through partnership between our burn center Certified Child Life Specialists (CCLS) and a burn quality nursing specialist with bedside pediatric experience to create a curriculum aimed at building confidence and providing a toolbox for bedside procedures such as wound care, intravenous line (IV) placement, and blood draws.

**Methods:**

An educational slide show was prepared, covering a wide range of topics including anticipated responses based on child developmental stage, and proper times to seek CCLS support. Emphasis was placed on addressing pain and fear experiences. The presentation was offered to day and night shifts 12 times between 9/2023-3/2024. All participants participated in a pre-survey at the beginning of each education session. Questions focused on assessment of confidence levels via Likert scale in addition to queries as to perceived benefits and challenges to implementation of comfort interventions. A post-survey is pending, following the opportunity to integrate lessons learned into clinical practice prior to reassessment.

**Results:**

Our pre-survey was completed by 25 burn nurses. Median confidence level reported for all assessed parameters, bedside procedures, use of comfort measures during procedures, and involvement of family was “very confident” (a 4 on a 5-point Likert scale). Nearly half of respondents reported no to moderate confidence with procedures, utilizing comfort strategies, and involving family members as support. The most cited barrier to implementation of comfort strategies during pediatric procedures was not knowing the right method to use (36.0%). Lack of comfort tools and distracting materials was also a common concern (32.0%).

**Conclusions:**

Implementation of this program has provided perceived added value to our nursing staff. While post-survey results are forthcoming, anecdotal feedback has been resoundingly positive. The CCLS team has subsequently been asked to expand the program to hospital-wide staff including the multidisciplinary emergency department team, burn service rotating residents, and burn center Advanced Practice Providers. We have also received significantly more requests for distraction supplies, for which we have applied for grants and partnered with the trauma program to expand our tangible offerings for children experiencing painful procedures.

**Applicability of Research to Practice:**

Improving the quality of Burn Center pediatric patient and parent experience is directly linked to increasing nursing staff comfort, which can be achieved through partnership with CCLS.

**Funding for the Study:**

N/A

